# HCV IRES-Mediated Core Expression in Zebrafish

**DOI:** 10.1371/journal.pone.0056985

**Published:** 2013-03-01

**Authors:** Ye Zhao, Wei Qin, Jing-Pu Zhang, Zhan-Ying Hu, Jun-Wei Tong, Cun-Bao Ding, Zong-Gen Peng, Li-Xun Zhao, Dan-Qing Song, Jian-Dong Jiang

**Affiliations:** 1 Institute of Medicinal Biotechnology, Chinese Academy of Medical Sciences and Peking Union Medical College, Beijing, China; 2 State Key Laboratory of Bioactive Substances and Functions of Natural Medicines, Institute of Materia Medica, Chinese Academy of Medical Sciences and Peking Union Medical College, Beijing, China; University of Modena & Reggio Emilia, Italy

## Abstract

The lack of small animal models for hepatitis C virus has impeded the discovery and development of anti-HCV drugs. HCV-IRES plays an important role in HCV gene expression, and is an attractive target for antiviral therapy. In this study, we report a zebrafish model with a biscistron expression construct that can co-transcribe GFP and HCV-core genes by human hepatic lipase promoter and zebrafish liver fatty acid binding protein enhancer. HCV core translation was designed mediated by HCV-IRES sequence and gfp was by a canonical cap-dependent mechanism. Results of fluorescence image and *in situ* hybridization indicate that expression of HCV core and GFP is liver-specific; RT-PCR and Western blotting show that both core and gfp expression are elevated in a time-dependent manner for both transcription and translation. It means that the HCV-IRES exerted its role in this zebrafish model. Furthermore, the liver-pathological impact associated with HCV-infection was detected by examination of gene markers and some of them were elevated, such as adiponectin receptor, heparanase, TGF-β, PDGF-α, etc. The model was used to evaluate three clinical drugs, ribavirin, IFNα-2b and vitamin B12. The results show that vitamin B12 inhibited core expression in mRNA and protein levels in dose-dependent manner, but failed to impact gfp expression. Also VB12 down-regulated some gene transcriptions involved in fat liver, liver fibrosis and HCV-associated pathological process in the larvae. It reveals that HCV-IRES responds to vitamin B12 sensitively in the zebrafish model. Ribavirin did not disturb core expression, hinting that HCV-IRES is not a target site of ribavirin. IFNα-2b was not active, which maybe resulted from its degradation in vivo for the long time. These findings demonstrate the feasibility of the zebrafish model for screening of anti-HCV drugs targeting to HCV-IRES. The zebrafish system provides a novel evidence of using zebrafish as a HCV model organism.

## Introduction

Hepatitis C virus (HCV) infection is one of the major causes of chronic hepatitis, which subsequently causes development of liver cirrhosis and hepatocellular carcinoma (HCC) [1]. Currently the main clinical therapeutic regimen is the combination of pegylated interferon (IFN-Peg) and ribavirin. However, the treatment is only partially effective [2]. Telaprevir and boceprevir, as peptidomimetic inhibitors of the HCV NS3/4A protease, have been approved for hepatitis C patients by US FDA. But these drugs have proved to cause drug-resistance in clinic [3]–[6]. Hence, discovery and development of novel anti-HCV agents continues to be an urgent need. The universal obstacle against drug discovery for HCV is the lack of adequate small animal models for HCV infection, replication and gene expression. To date, several model systems have been created for HCV infection research. For example, the chimpanzee model was proved to support the entire life cycle of HCV [7]; the mouse model with chimeric human livers can be infected with HCV, which was developed to study HCV life cycle in the absence of immune system [8]. These models have some limitations yet, such as ethics issues, complicated surgical procedures, high genetic variation of HCV isolates, poor reproducibility, low HCV viralemia, operational security, etc. which have hampered the application of these models in evaluating new drug candidates [9]. Therefore, development of small animal models for HCV will greatly facilitate the discovery and development of new anti-HCV drugs, with superiorities in *in vivo* environment, rapid and sensitive assay, low-cost, definite targets and early intervenes.

HCV is an enveloped virus with single-stranded positive-sense RNA, and belongs to the Flaviviridae family [10]. Its RNA genome of approximately 9600 nucleotides codes for a single polyprotein with about 3000 amino acids [11]. HCV polyprotein is proteolytically processed by both cellular and viral proteases into at least 10 individual proteins, including structural proteins (core, E1, E2 and p7) and non-structural proteins (NS2, NS3, NS4A, NS4B, NS5A, and NS5B) [11]. Among these proteins, core is recognized to be important in liver pathological process. It can affect the lipid metabolism pathway through promoting fat accumulation in hepatocyes [12]–[14], participate oxidative stress and apoptosis [15]–[17], steatosis [15], liver fibrosis [16], and hepatocellular carcinoma (HCC) [17], [18]. There are two untranslated regions (UTR) at both N and C terminals of HCV genome. The IRES at 5′ UTR mediates viral protein expression by directly recruiting the ribosome 40s subunit to the starting site of the genome [19], which is different from the cap-dependent mechanism. It is confirmed that IRES sequence between nt 42 and 372 is essential for mediating HCV translation activity [20] and is more conserved than HCV cds. Therefore, the compound targets specifically to HCV IRES might repress the viral protein translation and have anti-HCV effects. At the moment, most of the anti-HCV drugs research focus on suppression of HCV replication enzymes. An *in vivo* model for HCV IRES function may add weight for new drug research on HCV.

The zebrafish has been well recognized as a useful model organism for studies of vertebrate physiology and human diseases [21], probably because it offers several unique advantages, e.g. genetic homology to humans [22], its transparent embryonic body, rapid development, a large number of reproductions, and feasibility of live imaging with fluorescent labeled internal organs. Zebrafish liver develops since 50 hours post fertilization [23], rendering a chance to observe hepatic changes and to image fluorescent signal if fluorescent protein expressed at young larvae. Transgenic zebrafish models with Hepatitis B virus X protein exhibited liver injury with lipid accumulation and steatosis-associated pathogenic change [24]. HCV core protein along with TAA (thioacetamide) could induce HCC in zebrafish in a shorter period of time compared with mice models [25]. In our previous study, we developed a zebrafish model of HCV sub-replicon. The model can mimic HCV replication mechanism with HCV RNA polymerase and 3′UTR sequence-dependant RNA amplification. Evaluation of two clinic drugs confirmed the model in its efficiency [26]. These studies demonstrate that zebrafish has the potential to play an important role in establishing liver disease models. In the present study, we established an HCV-expression model in zebrafish liver, through the IRES-mediated HCV core translation. This HCV-zebrafish model can be used to discover anti-HCV agents that target IRES-mediated HCV gene expression which is another significant process in HCV life.

## Results

### HCV IRES Activity and HCV Core Expression in Zebrafish Liver

**Figure 1:**
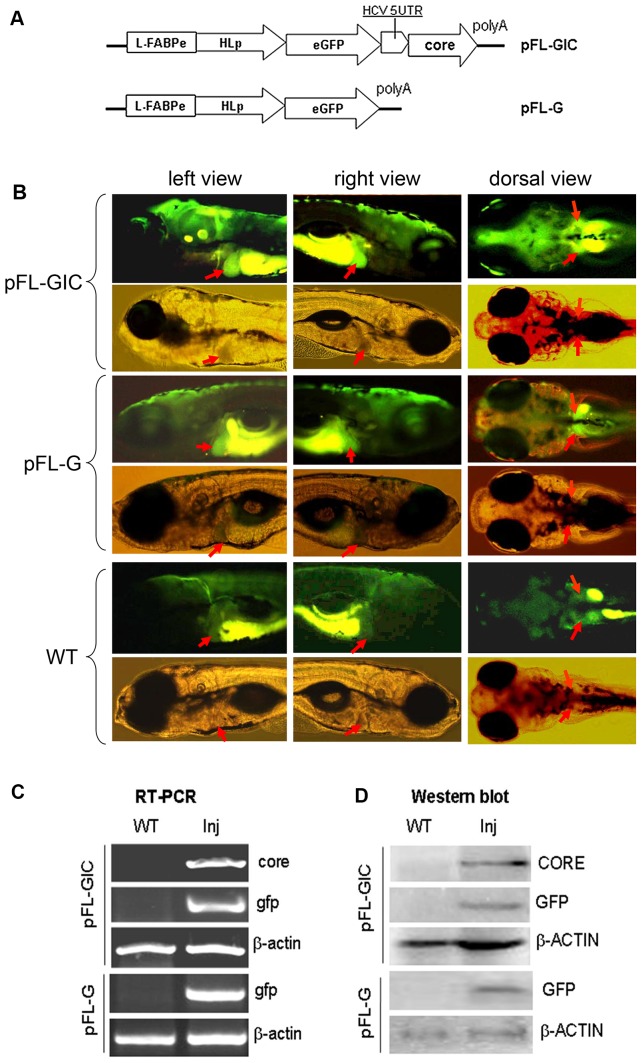
Expression of Core and GFP in zebrafish larvae. A. Diagrams of plasmid constructs. In pFL-GIC the core cds and GFP cds are driven by L-FABP enhancer and HL promoters, and separated by HCV IRES residing between them. pFL-G was a control construct without HCV IRES-core sequence. B. Observation of expression of GFP in 8-dpf zebrafish larvae under a flourescence microscopy. In each group, upper panel shows larvae images under the GFP excitation filter; lower panel shows the same larvae under visible light. Positive bright green fluorescence was seen in liver of the larvae injected with pFL-GIC or pFL-G, but in WT larvae only the auto-fluorescence appeared with yellowish fluorescence. Red Arrows indicate liver region in the larvae. A GFP filter (480 nm excitation, 505 nm emission) were used to excite the EGFP (Green). Original images were 40×. C. RT-PCR assay for transcription of core and gfp in pFL-GIC injected larvae, compared to that of pFL-G injection and that of wildtype larvae; β-actin was used as a loading control. All the larvae in this assay were collected at 10 dpf. D. Western blotting Assay for CORE and GFP proteins in pFL-GIC injected larvae, compared to that of pFL-G injection and that of wildtype larvae; β-ACTIN was used as a loading control. All the larvae in this assay were collected at 10 dpf.

**Figure 2:**
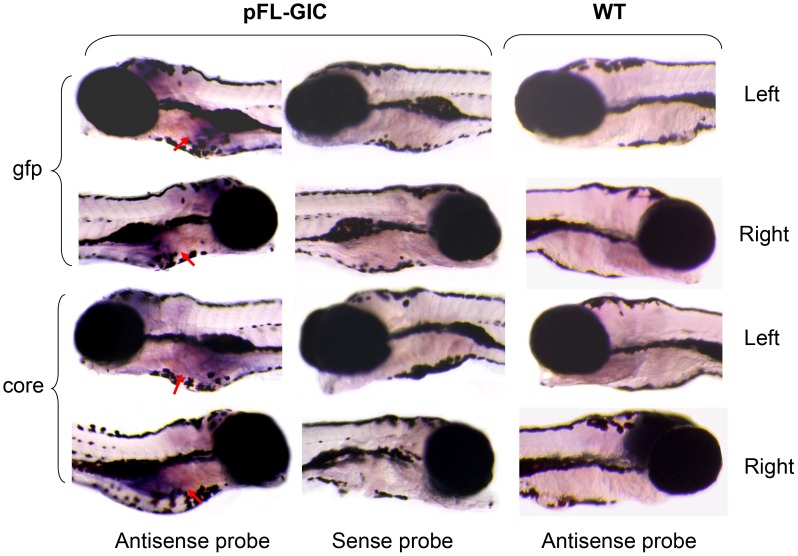
HCV core and gfp expression in zebrafish liver. Whole mount *in situ* hybridization was carried out on 8-dpf larvae using antisense or sense RNA probes of core and gfp, respectively. The core and gfp signals were mainly occurred in liver area in pFL-GIC injected larvae at 8-dpf with antisense probes (red arrows), and were not seen in WT larvae and in the sense probe group with pFL-GIC injection as a negative control. Original images were 40×.

The expression of gfp and HCV core genes was designed to be co-transcribed by human hepatic lipase promoter and zebrafish L-fabp enhancer in zebrafish liver [27], [28]. Firstly, bright GFP green fluorescent signal was detected under a fluorescence microscopy and obviously localized in liver area in 8-dpf larvae injected either with pFL-GIC or with pFL-G control construct (Fig. 1B). Spontaneous fluorescence was seen in the body as well with yellowish fluorescence in wildtype larvae. Then the expression of gfp and core genes was verified with RT-PCR and Western blotting, and both assays demonstrated positive results in injected larvae at 10-dpf in both mRNA and protein levels (Fig. 1C, 1D). To confirm liver-selective expression of core and gfp, Whole mount *in situ* hybridization (WISH) was done to detect the both mRNA. As shown in Fig. 2, the signals were localized in liver area of the 8-dpf larvae injected with pFL-GIC, consistent with the green fluorescence detection.

**Figure 3:**
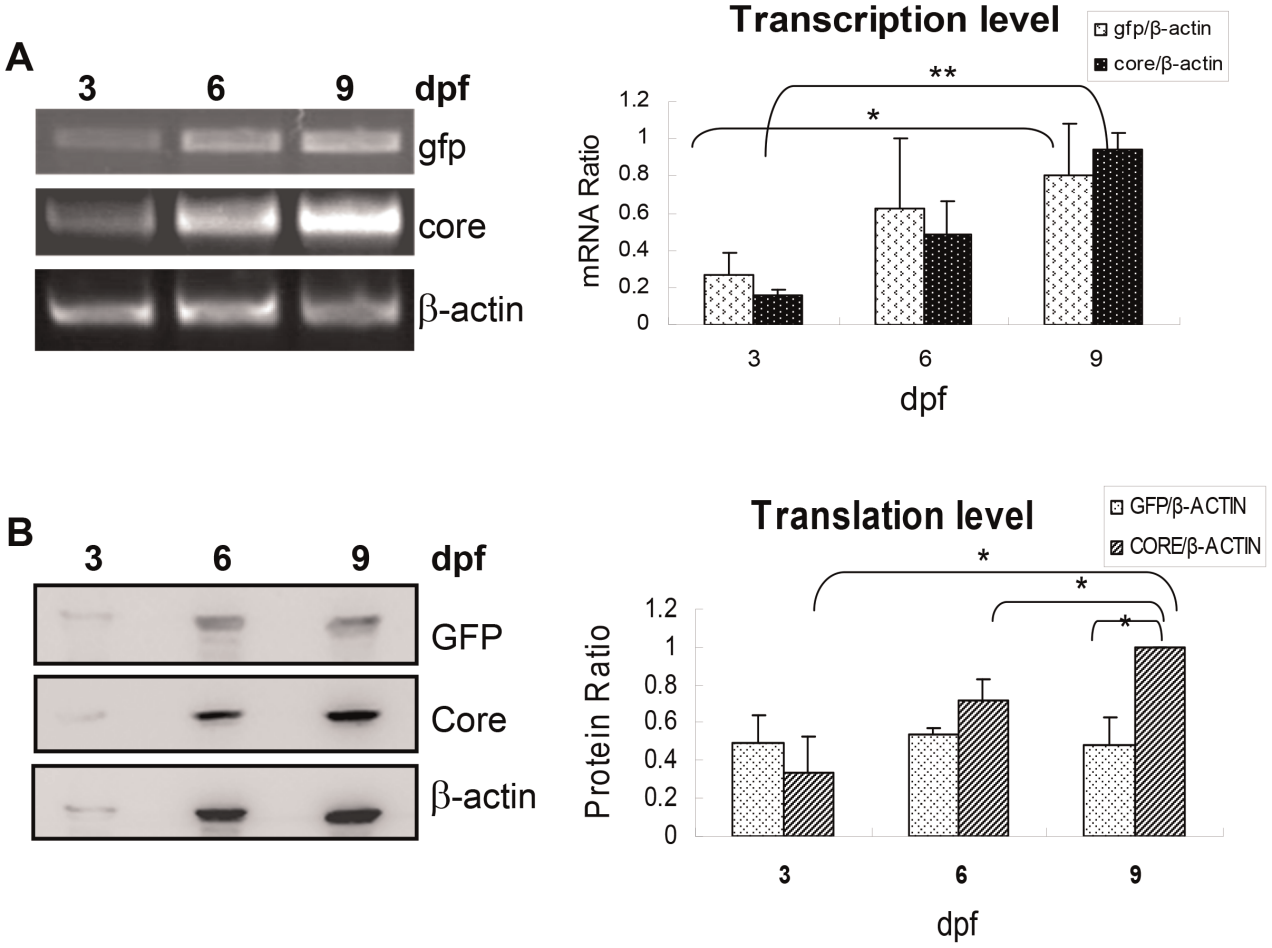
Time-dependent expression of HCV core and gfp during the early larva stage. A. Transcription level of core and gfp in pFL-GIC injected zebrafish larvae at 3-, 6- and 9-dpf was examined by RT-PCR. β-actin was used as a loading control. The band semiquantitative density scanning was done and normalized by β-actin signal for their transcriptional level evaluation (the right histogram. * p<0.05, ** p<0.01). B. Translation level of CORE and GFP in pFL-GIC injected larvae was detected with Western blotting at 3-, 6- and 9-dpf. β-ACTIN was used as a loading control. The bands of CORE and GFP were scanned and normalized by β-ACTIN signal for their protein level evaluation (the right histogram. * p<0.05).

To view the expression of HCV core gene mediated via HCV IRES and that of GFP via a canonical capped mRNA mechanism in pFL-GIC construct, time-dependent transcription and translation of the HCV IRES-core and gfp were examined by RT-PCR and Western blotting. At 3, 6, 9 days post fertilization, core and GFP transcriptions increased along with time (Fig. 3A). Also, the CORE protein went up more markedly than GFP in a time-dependant manner (Fig. 3B), suggesting that HCV IRES is functional in zebrafish liver.

### Safety and Pathological Impact in pFL-GIC Injected Zebrafish Larvae

**Figure 4:**
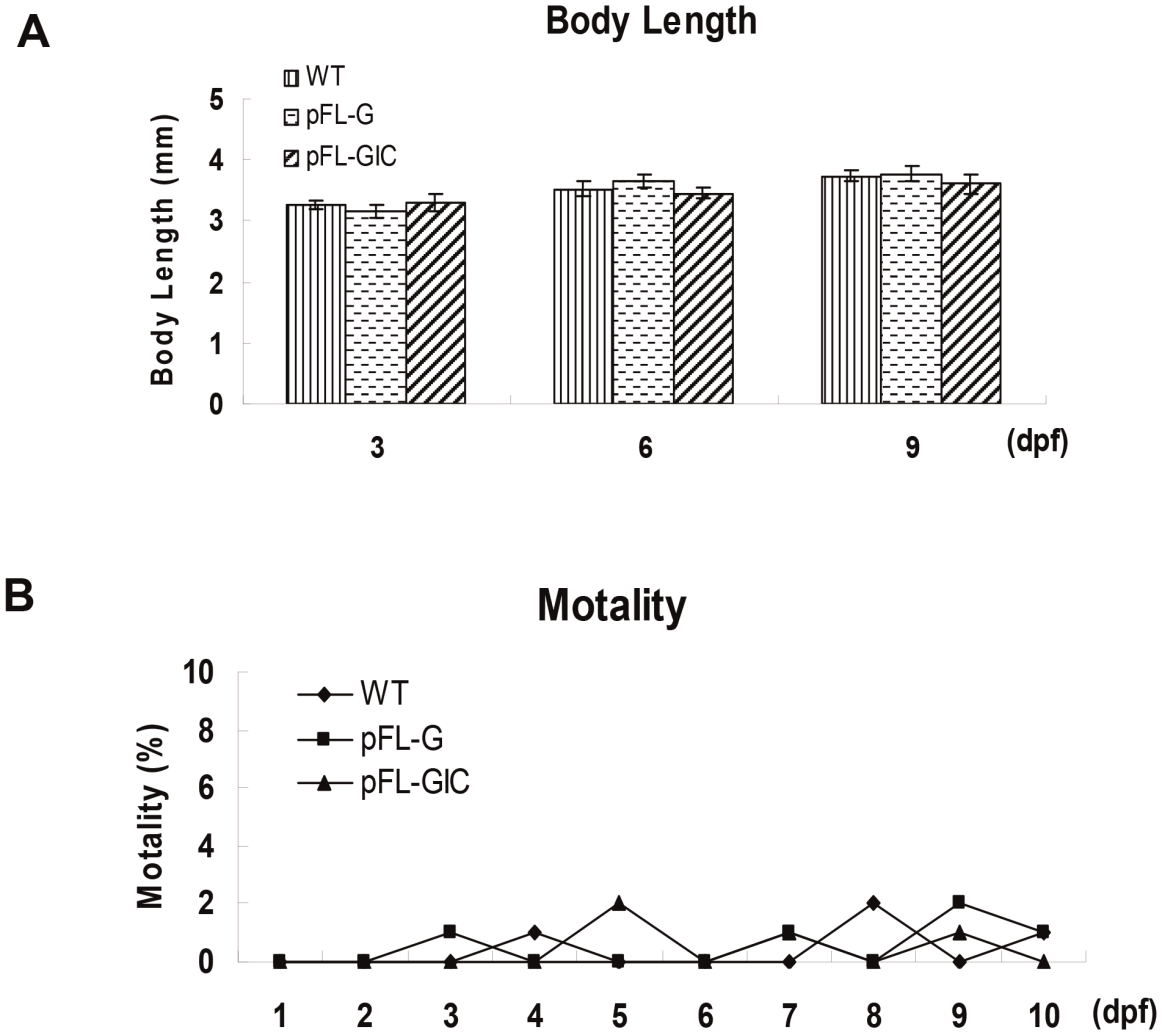
Biological impact on development in zebrafish larvae after pFL-GIC injection. A. The body length of pFL-GIC injected zebrafish larvae, compared with pFL-G injected zebrafish and WT larvae at 3-, 6- and 9-dpf (p>0.05). B. The mortality curve of zebrafish larvae after pFL-GIC injection, compared to that of pFL-G injected zebrafish and that of WT in the first 9 days during the embryonic development (p>0.05).

The toxic effect in pFL-GIC injection larvae was assessed by measuring body length and mortality in comparison with that of wild type larvae as well as pFL-G control injection. As shown in Figure 4A, expression of HCV core protein did not slow down the growth of the larvae (p>0.05) at least in the first 9 days of embryonic development; deformity phenotypes were not observed as well (data not shown). Difference in mortality was not detected among the three groups, pFL-GIC, pFL-G and wildtype larvae during the development from zygote to larva (10 dpf) (Fig. 4B), suggesting that the HCV construct injection as well as the subsequent HCV-core gene expression had no toxicity to embryonic development in zebrafish.

**Figure 5:**
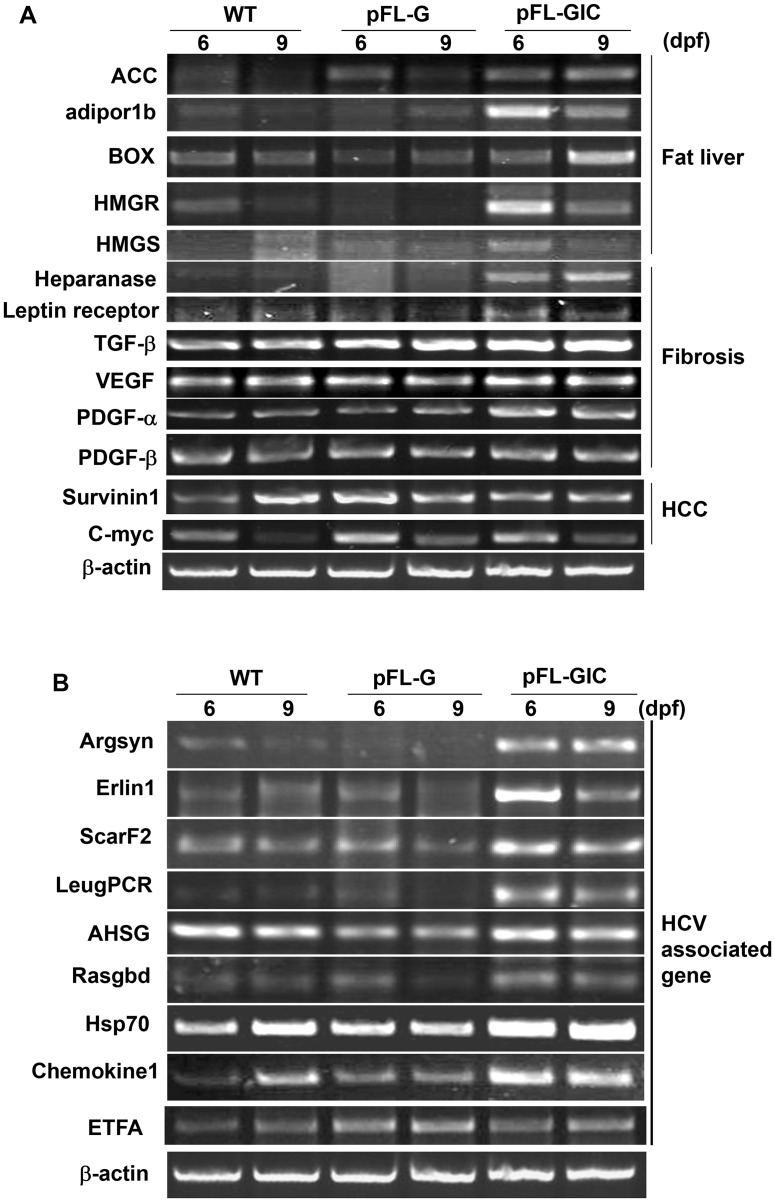
Expression of liver pathological marker genes responded to HCV core expression in the HCV-zebrafish model. RT-PCR was used to assess the gene expression in pFL-GIC- and pFL-G-injected larvae and in WT larvae at 6-dpf and 9-dpf. A. Result of genes involved in fat liver, steatohepatitis and fibrosis; B. HCV-infection associated genes. WT as a normal control and pFL-G as a vehicle control injection.

To investigate whether expression of pFL-GIC construct results in HCV-related pathological change, we examined the expression of several pathological biomarker genes, which might correlated to various stages of liver injury such as fat liver, steatohepatitis, liver fibrosis and hepatocellular carcinoma (HCC). The measurement was done with RT-PCR for 6-dpf and 9-dpf larvae, in comparison with that of wild type larvae and control pFL-G larvae. As shown in Figure 5A, expression of some of the hepatopathy-related genes were elevated such as acetyl-CoA carboxylase (ACC), adiponectin receptor 1b (adipor1b), heparanase, TGF-β, PDGF-α, HMG-CoA synthase (HMGS), HMG-CoA reductase (HMGR) and branched-chain acyl-CoA oxidase (BOX), indicating a risk of developing fatty liver and liver fibrosis in the core-positive larvae. The control pFL-G larvae exhibited a gene expression pattern similar to that in wildtype. The results were consistent with previous reports in other models [29], [30]. The expression of the tumor biomarkers (Survinin1 and C-myc) remained unchanged at the transcription level, probably reflecting a pathological stage prior to carcinogenesis in 9 dpf larvae in this model.

To validate the relationship with HCV infection, a panel of HCV-infection gene markers was examined. Out of the nine study genes, seven gene expression such as argsyn, scarF2, rasgbd, chempkine1, leugPCR, hsp70 and erlin1 were increased remarkably; while ETAF (electron transfer flavoprotein alpha polypeptide) and ASHG (Alpha 2-HS-glycoprotein) expression remained unchanged (Fig. 5B). The results suggest that the pFL-GIC injected zabrfish larvae may mimic, at least partly, the HCV-caused pathological change at gene level.

### Evaluating Anti-HCV Drugs using HCV-zebrafish Model

**Figure 6:**
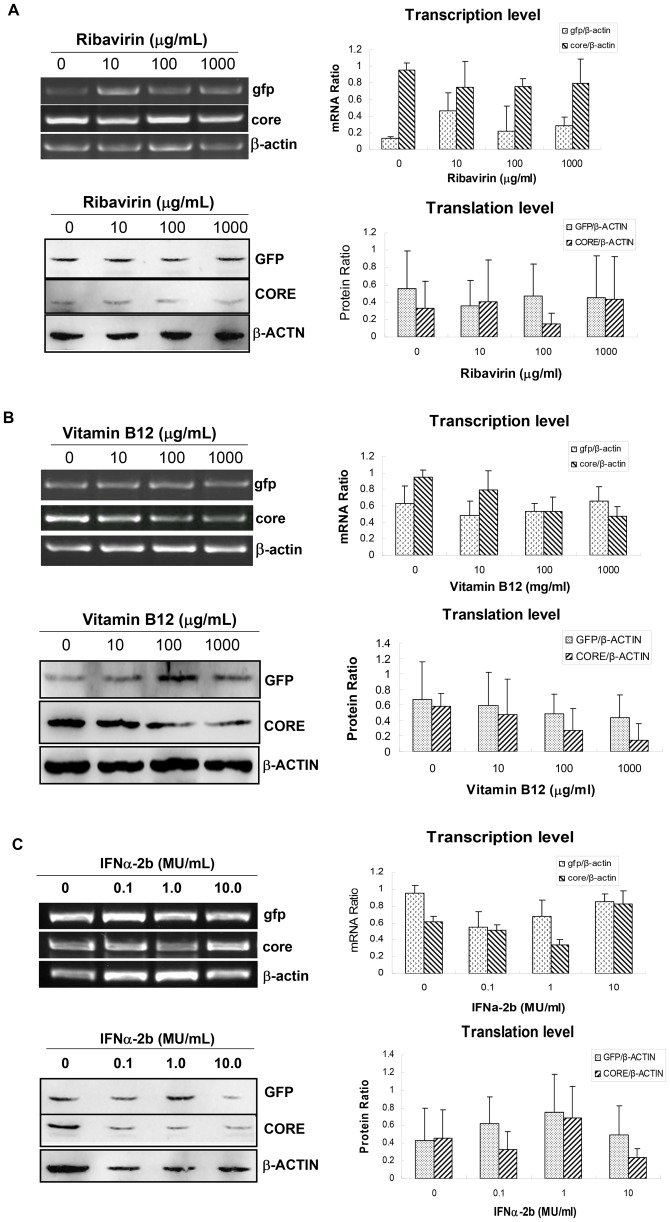
Verification for the efficiency of the HCV IRES-mediating expression zebrafish model with anti-HCV drugs. RT-PCR (upper panel) and Western blotting (lower panel) were used for detecting expression of core and gfp in pFL-GIC-injected larvae that were exposed to ribavirin (A) and vitamin B12 (B) drugs at gradient concentrations from 5-dpf to 10 dpf. C. Result of IFNα-2b co-injected with plasmid pFL-GIC. All the larvae were collected at 10-dpf for RT-PCR and Western blotting assays. Untreated larvae were as a control. Both cDNA and protein bands were scanned against β-actin cDNA or β-ACTIN protein respectively for semiquantitative evaluation of core and gfp expression (right histograms).

**Figure 7:**
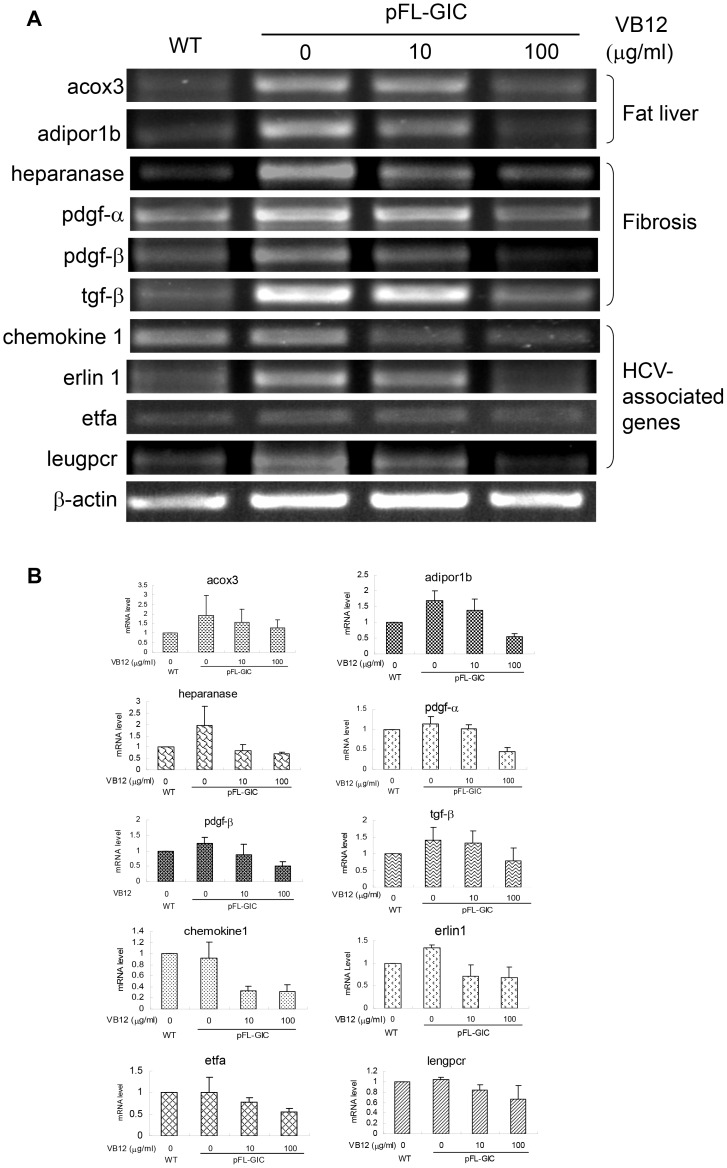
Down-regulation of liver pathological gene mRNA levels by vitamin B12 exposure. Zebrafish larvae with pFL-GIC injection were exposed in vitamin B12 from 5 dpf to 8 dpf, and collected for detection of the pathological gene mRNA levels by RT-PCR. Target gene bands were scanned against β-actin band for semiquantitative evaluation. WT used as a normal control. A. Representative mRNA levels of genes involved in fat liver, fibrosis and HCV-associated genes; B. Histograms for relative variation of mRNA levels that were normalized to WT.

To learn whether this HCV-zebrafish model was suitable for the efficacy evaluation of anti-HCV drugs that target HCV IRES, the pFL-GIC injected larvae at 5dpf were treated with ribavirin or vitamin B12, respectively, for another five days. In addition, another drug IFNα-2b was coinjected with pFL-GIC into embryos. Then, core and gfp were examined in the 10-dpf larvae at both transcription and translation levels. As shown in Figure 6B, the expression of HCV core gene was significantly reduced in a dose-dependent manner in the larvae incubated in the vitamin B12-containing water; however, the expression of GFP gene was not affected apparently, indicating that HCV IRES mediated core expression probably was inhibited by the drug. It is also observed that the transcription level of core was also decreased in a dose-dependent manner comparing with gfp group. The result hints that vitamin B12 might have certain degree of interaction with HCV IRES. The similar result was not observed in ribavirin and IFNα-2b treated groups (Fig. 6A, 6C), suggesting that ribavirin might have different mechanisms from vitamin B12 in its action against HCV; and IFNα-2b inefficiency may be resulted from its degradation in vivo for the long time since once injection at one-cell stage, which was proved by western blotting with IFNα-2b antibody (data not shown). Further, in order to confirm and evaluate vitamin B12 action in the HCV-IRES zebrafish model, mRNA levels of some gene markers involved in Fat Livers (adipor1b and acox3), Fibrosis (heparanase, pdgf-α, pdgf-β and tgf-β) and HCV infection (chemekine 1, erlin 1, etfa and lengpcr) were examined by RT-PCR in the larvae that were treated by pFL-GIC injection and vitamin B12 exposure. The results indicated that vitamin B12 exposure indeed down-regulated the gene mRNA levels which were elevated in pFL-GIC injected larvae (Fig. 7). Thus, we consider this zebrafish system a suitable small animal model to evaluate anti-HCV drugs that work through inhibition of HCV IRES.

## Discussion

Although HCV genome has a high mutation rate, the IRES sequence is relatively conservative among HCV genotypes [31]. Therefore, HCV IRES as well as its translation initiation complexes are attractive drug targets. Early studies demonstrated that the inclusion of nt12 to nt30 of the core protein coding sequence was essential for an efficient IRES activity [32]. Thus, in our construct design, the whole core coding sequence was contained both as a reporter and a pathogenic factor in the zebrafish model. Though it was reported the core is an inhibitor on HCV IRES [33], [34], our results show that the inhibition can be ignored in this research.

IRES activity can be modulated by a number of proteins or oligonucleotides [35]–[38]. HCV IRES-dependent translation also could be inhibited by vitamin B12 *in vitro* [39], [40]. Vitamin B12 selectively inhibited HCV IRES-dependent translation with no effect on cap-dependent translation in a dose-dependent manner; the domain IV of IRES is the responsible element for this inhibition [40]. The toeprinting data also has strongly implied that vitamin B12 binds directly to the HCV IRES RNA and prevents the 80S complex from leaving the start site [41]. The precise mechanism of Vitamin B12 has not been clarified clearly yet. Previous study revealed that domain II of the HCV IRES played a crucial role as the apical hairpin loop, interacting with eIF5 to facilitate eIF2a-GTP hydrolysis, and leading to eIF2a-GDP release and subsequent 80S ribosomal assembly [42], [43]. Benzimidazole was screened out to bind the subdomain IIa of HCV IRES. It could inhibit IRES-mediated translation in HCV-infected cells through a conformational induction of a widened inter helical angle in subdomain IIa which facilitated the undocking of subdomain IIb from the ribosome [44], [45]. Interestingly, vitaminB12 contains the structure of benzimidazole subunit. So we raise a hypotheses that vitaminB12 may play an inhibitory role as a benzimidazole analogue and modulate the conformation of the HCV IRES. Our study demonstrated that vitamin B12 appeared to inhibit both transcription and translation of the HCV IRES-mediated gene expression *in vivo*, compared with GFP expression (Figure 3) in time-dependency. It indicates that HCV IRES plays a significant role not only in regulation of translation level but also in transcription level. Our result is coincidental with the previous study which demonstrated that HCV IRES presented strong promoter activity in both HuH7 and HeLa cells [46]. However, the IRES promoter mechanism remains to be investigated. IFN-α was reported to have a role of inhibiting HCV replication by targeting IRES site in cell level [47]. In this study we did not observe the action of IFNα-2b. It may be too long to keep its activity for a foreign protein in zebrafish with once injection at one-cell stage; or probably HCV-IRES is not a target of IFNα-2b. Another antiviral drug ribavirin also showed no activity on the zebrafish model. It is reasonable that ribavirin function in inhibition of viral genomic replication, not for HCV gene expression. Our previous study demonstrated ribavirin suppression role on HCV subreplicon amplification in zebrafish model [26].

HCV core gene is known to play crucial roles in lipid metabolism, HCV-induced steatosis and HCC [13], [15], [36]. In this study, in the HCV IRES-zebrafish model the remarkably increased expression in the steatosis marker genes, like heparanase, adiponecin, TGF-β, PDGF-α, HMGR may be related to the effect of core in hepatocytes. Synthesis and metabolism of cholesterol is controlled by several enzymes including HMGR and HMGS in hepatocytes [30]. Therefore, it is inferred that HCV core may be complicated in cholesterol metabolic pathways to affect lipid metabolism in host cells. TGF-β and PDGF-α are known as pro-fibration facors; and argsyn, scarF2, Erlin1 and Hsp70 are correlated to HCV infection [37], [38]. In this study, the elevations of these genes consist with the results in mice models reported before. It suggests that the zebrafish model of pFL-GIC for HCV expression seems to be successful and could mimic mammal hepatic steatosis and fibrosis at gene levels. Further, vitamin B12 action on these gene transcription levels was confirmed with down-regulation role, which provided substantial evidence of VB12 suppression at HCV pathology process in the zebrafish model. Our results suggest that this zebrafish model can be used to screen compounds with HCV IRES-targeted antiviral mechanism.

In summary, we have demonstrated HCV-IRES functionality in zebrafish and the HCV-IRES zebrafish model a useful tool for anti-HCV drug evaluation.

## Materials and Methods

### Plasmids

HCV 1b (J4L6s) strain (accession no. AF054247) was provided by Dr. HS Chen (Institute of Medicinal Biotechnology, Beijing). Human cell line L02 [48] was supplied by PLA Key Laboratory of Experimental Hematology/Department of Experimental Hematology, Institute of Radiation Medicine, Military Medical Academy of Sciences. To have liver expression specificity of the gene construct, human hepatic lipase promoter sequence (hHLp, 685 bp) [27] was cloned from the human cell line L02. Zebrafish liver fatty acid binding protein (zL-FABP) enhancer sequence 435 bp was synthesized by Sangon Biotech (Shanghai) Co. Ltd. based on the GenBank No. AF512998 [28]. Both of the sequences were validated with sequencing.

Gene construct pFL-GIC was generated by insertion of HCV IRES-core sequence at downstream of egfp in the pEGFP-C2; CMV promoter of pEGFP-N1 was substituted by hHL promoter linking with zL-FABP enhancer at its 5′upstream. Then, DNA coding sequence of egfp and HCV IRES-core was digested with BsrGI and RsrII and substitute egfp sequence at the same site of pEGFP-N1 (Fig. 1A). In this construct, the first cistron (encoding GFP) is translated via a canonical cap-dependent mechanism. While the second cistron (encoding HCV core) is translated only mediated by HCV IRES sequence. Both GFP and core protein should have liver-specific expression under the control of hHL promoter and zL-FABP enhancer. For a control construct, pFL-G, the CMV promoter of pEGFP-N1 was substituted by hHL promoter and zL-FABP enhancer (Fig. 1A). Both vectors were linearized by RsrII for injection.

### Microinjection and Fluorescent Microscopic Examination

Adult zebrafish (Danio rerio) AB line were a gift from Dr. Anming Meng (Tsinghua University, Beijing, China). The zebrafish were incubated in a controlled condition of 14-h light/10-h dark cycle at 28±2°C. The fragment pFL-GIC or pFL-G was injected into 1–8 cell-stage embryos at a concentration of 1 ng/µl. Green fluorescence positive larvae were examined from 6 to 9 days post fertilization (dpf), using fluorescence microscopy (Olympus IX51) with GFP wave length (480 nm excitation, 505 nm emission).

### RT-PCR Analysis

The mRNAs of HCV Core and GFP were detected by reverse transcription-PCR (RT-PCR). Total RNA of larval zebrafish was harvested with Trizol Regent (Invitrogen, China). The first strand of cDNA was synthesized from 1 µg of the total RNA using AMV reverse transcriptase (Promega, US). After the reverse transcription reaction, the cDNA template was amplified by polymerase chain reaction with Taq polymerase (TaKaRa, Japan). Then PCR was performed with 1.0 µl cDNA that was properly diluted based on preliminary test and 0.5 µl 50 µM primer pairs, using a program of 96°C, for 5 min, 94°C for 40s, 50°C for 40s and 72°C for 40s, 30 cycles for the potential pathological genes, 25 cycles for β-actin; the reaction mixtures were incubated at 72°C for an additional 10 min to allow a complete synthesis. The RT-PCR products were subjected to 1.0% agarose gel electrophoresis. β-actin was used as loading control. Then the core and gfp bands were scanned for their relative transcription level evaluation against β-actin, in time-dependent test during larva development and drug treating experiments.

The PCR primers for the amplification of the fatty liver associated genes, acetyl-CoA carboxylase (ACC), and adiponectin recptor 1b, the fibrosis associated genes, Heparanase, Leptin receptor, TGF-β, VEGF, PDGF-α and PDGF-β, the lipid biosynthesis (Steatohepatitis) genes, such as acyl-Coenzyme A oxidase 3 (acox3), branched-chain acyl-CoA oxidase (BOX), HMG-CoA synthase (HMGS), HMG-CoA reductase (HMGR), the HCC (hepatocellular carcinoma) marker genes, survinin1, C-myc, and the HCV associated marker genes, argininosuccinate synthetase (Argsyn), ER lipid raft associated 1 (Erlin1), solute carrier family 2 (ScarF2), leucine rich repeat containing G protein coupled receptor (LeugPCR), alpha 2-HS-glycoprotein (AHSG), ras-related GTP binding D (Rasgbd), heat shock cognate 70 (Hsp70), chemokine (C–C motif) ligand 1 (Chemokine1), electron transfer flavoprotein alpha polypeptide (ETFA), and β-actin are listed in Table 1. The results were normalized to that of β-actin.

**Table 1:** The PCR primers for the amplification of pathology genes.

Gene	Primer sequence (5′ - 3′)
Chemokine-1	F: TCTCTTCTCACCTGCCCTAAR: ATTGCTTGCACCTTCTCCCTC
AHSG	F: GGAAGGCAGCGGTGAAAR: ATGGTCTGGCCCGAGTG
Hsp70	F: GCGACACCTCTGGAAACR: TGCTCAGCCTGCCCTTG
Rasgbd	F: ATCCCTCAACTTCCCACCR: TCTGCCTGCTCCACCTC
Argsyn	F: GACAGGACGAGGACTTTGR: TGACGGGAACAGGAATG
ScarF2	F: CTCTTGCGTCTACAGGGR: GCTCAGCGGTTTCTATT
Leugpcr	F: GGTGTTTGTCTGGGTTGR: GGTCTGAGTGAAGAGGGA
BOX	F: GCCTGGGTTGGTTAGAAGR: GCGGCGGATGACCGAGTA
HMGS	F: GACGGTCGTTACGCTCTGGTTGTR: CTGTCAAAATAAGTGTCCTCAATC
HMGR	F: GGGTTCGCAGTGATAAAGGAR: TTGCTGAGGTAGTAGGTTGGTC
Heparanase	F: CAAGCGTTTAGTCACTCTGGCR: GGTTGCATTCCACGAGTTGTC
Leptin receptor	F: GTCACACTGATGATGTCACAGAACCAGATGR: GCTAAAGACCTCTATTACCTCGAGATGACC
Survinin1	F: ATTTCCACACCAACCTCCCACR: CGAAAGGAAAAGAGCGAGGTC
C-myc	F: CCCAGCCGGAGACAGTCGCTCTCCACCGCGR: CCACAGTCACCACATCAATTTCTTCCTCC
Erlin-1	F: CAGGCTGCTCATCACCGR: AGGCTTTGGGCTTGGAC
Adipor1b	F: ATGGGAGGGACATTGGCGAGTTATR: TGAGTAGTAGAGCCACGGAACGAA
TGF-β	F: ACCAGCAGAGCACGGATAAR: GGCAGAGGGTCAAGGATT
VEGF	F: ACTGGAATTACAATCATGR: ATCATCTTGGCTTTTCAC
PDGF-α	F: CCACAGGGGAGATGAGAAR: TATCTGGTGCCACGCTCA
PDGF-β	F: GGACCCTCTTCCTCATCTCR: TGGGACACGTAACTGACAGC
ETFA	F: GAAAGCGTGAAGGTGTTR: GATGGCTACGATGGTCT
ACC	F: TTAGACCTGGATCAACGGCGR: CATGATCTGTCCTGTACGGG
acox3	F: CATATGAGGGAGACAACAACGTR: TGAGAGTCCACAAGCCATAGAG

### Whole Mount *in situ* Hybridization (WISH)

Core (nt 430–702) sequence and GFP gene were used as templates for hybridization antisense RNA probe synthesis, using DIG RNA Labeling Kit (Roche Diagnostics Scandinavia AB, Bromma, Sweden). Larval zebrafish of 8-dpf were fixed with 4% paraformaldehyde for 10 hrs at 4°C and washed with 1×PBST, and permeabilized before being soaked in the probe. The larvae were treated with proteinase K and DNase I separately, pre-hybridized in 65°C for 4 hrs, and hybridized with the RNA probe at 65°C overnight. The residual probe was washed with 0.2×SSC, followed by incubating with anti-Dig-AP (Roche) at 4°C over night. After wash with 1×PBST, the samples were colorized with BCIP/NBT for 30 min and stopped with 1×PBST washing [49]. Stained whole-mount larvae were observed in glycerol and visualized under an Olympus SZX16 stereomicroscope. Pictures were taken with a Canon 450D camera.

### Western Blotting

Larval zebrafish proteins were extracted with lysis buffer and separated in the 12% SDS-polyacrylamide gel electrophoresis (PAGE). The protein bands were transferred onto a nitrocellulose membrane followed by blocking of the membrane with TBS containing 10% skim milk. The membranes were incubated with mouse anti-HCV core antibody (1∶500 dilutions, MA1-080; Thermo Fisher Scientific Inc.) or rabbit anti-GFP antibody (sc-8334; Santa Cruz Biotechnology, Inc.) at 1∶2000 dilutions in TBS containing 1% skim milk; then the membrane was washed and incubated with secondary antibodies, HRP-conjugated goat anti-mouse or goat anti-rabbit IgGs (1∶2000 dilutions, Zhongshanjinqiao Co. China) for 2 hrs at RT. Chemiluminescent signals were detected using the Supersignal® West Pico chemiluminescent substrate (Thermo) with AlphaEase® FC Imaging System (Alpha Innotech Corporation).

### Drug Treatments

VitaminB12, ribavirin and IFNα-2b were from the National Institute for the Control of Pharmaceutical and Biological Products, Beijing, China. For inhibition of HCV core expression, Vitamin B12 and ribavirin at final concentration of 1000, 100 or 10 µg/ml, was added to zebrafish cultivation water, respectively. The zebrafish embryos injected with or without the gene construct were incubated in the drug-containing water from 5 dpf to 10 dpf, and then the larvae were collected for the next test. As vitamin B12 was light-sensitive, hood was used after adding vitamin B12 into the incubation water. IFNα-2b at concentration of 0.1, 1.0 and 10 MU/mL respectively was co-injected with the pFL-GIC construct before 8-cell stage. About 50 larval zebrafish or embryos were collected from each treatment group for RT-PCR or Western blotting. For detection of down-regulation of liver pathological gene mRNA levels by vitamin B12 exposure, zebrafish larvae were exposed in vitamin B12 at final concentration of 100 and 10 µg/ml respectively from 5 dpf to 8 dpf, then collected for RT-PCR detection.

### Statistical Analysis

Data in bars represent mean ± s.d in histograms. The means and standard deviations are derived from at least triplicates. Statistical analyses were performed using Oneway ANOVA Tests and P-values, 0.05 were considered as significant. The zebrafish larvae used in the study were randomly selected.

### Ethics Statement

This study was carried out in strict accordance with the recommendations in the Regulation for the Management of Laboratory Animals of the Ministry of Science and Technology of China. The protocol was approved by the Committee on the Ethics of Animal Experiments of the Institute of Medicinal Biotechnology, Chinese Academy of Medical Sciences (IMBF20060302).
